# The Application of Whole−Exome Sequencing in Patients With FUO

**DOI:** 10.3389/fcimb.2021.783568

**Published:** 2022-01-12

**Authors:** Wanru Guo, Xuewen Feng, Ming Hu, Yanwan Shangguan, Jiafeng Xia, Wenjuan Hu, Xiaomeng Li, Zunjing Zhang, Yunzhen Shi, Kaijin Xu

**Affiliations:** ^1^State Key Laboratory for Diagnosis and Treatment of Infectious Diseases, National Clinical Research Center for Infectious Diseases, Collaborative Innovation Center for Diagnosis and Treatment of Infectious Diseases, The First Affiliated Hospital, College of Medicine, Zhejiang University, Hangzhou, China; ^2^Department of Respiratory Medicine, Lishui Hospital of Traditional Chinese Medicine (TCM), Lishui, China; ^3^Department of Infectious Diseases，Dongyang People’s Hospital, Dongyang, China

**Keywords:** next-generation sequencing, whole exome sequencing, fever of unknown origin, gene mutation, genetic diagnosis

## Abstract

**Background:**

Fever of unknown origin (FUO) is still a challenge for clinicians. Next-generation sequencing technologies, such as whole exome sequencing (WES), can be used to identify genetic defects in patients and assist in diagnosis. In this study, we investigated the application of WES in individuals with FUO.

**Methods:**

We performed whole-exome sequencing on 15 FUO patients. Clinical information was extracted from the hospital information system.

**Results:**

In 7/15 samples, we found positive results, including potentially causative mutations across eight different genes: *CFTR*, *CD209*, *IRF2BP2, ADGRV 1*, *TYK2*, *MEFV*, *THBD* and *GATA2*.

**Conclusions:**

Our results show that whole-exome sequencing can promote the genetic diagnosis and treatment of patients with FUO.

## Introduction

Fever of unknown origin (FUO) has been a clinical problem since it was defined in 1961. FUO was first proposed by Petersdorf and Beeson in 1961, and was defined as a fever lasting more than 3 weeks with a temperature over 38.3°C on several occasions, that remained undiagnosis after one week of inpatient investigation (Petersdorf and Beeson, 1961). Accurate diagnosis can be challenging because a wide variety of diseases, including some rare diseases, can cause fever. Current methods including laboratory examinations, radiographic modalities and invasive investigations, are of limited use and sometimes cannot fully explain the disease and pathogenesis. Since infectious diseases are still the leading cause of FUO, many studies in recent years have focused on the etiology of the disease, and new technologies such as metagenomic next-generation sequencing (mNGS) have been applied to detect pathogens (Gu et al., 2019; Wright et al., 2021). However, few studies have focused on the role of host factors, which also play an important role in the development of diseases. It is novel for clinicians to explore the causes of fever from the perspective of host factors. Studies have shown that although phenotypic expression is highly variable, some cases of fever are related to gene mutations (Özen et al., 2017). For example, systemic autoinflammatory syndrome is known to often manifest as fever of unknown origin and is diagnosed by clinical suspicion and genetic testing (Lachmann, 2015). Some autoimmune diseases are also considered to be due to genetic factors (Liu et al., 2015; Erman and Çipe, 2020). Next-generation sequencing technologies (NGS), such as whole exome sequencing (WES), can be used for diagnosis or research to identify genetic mutations associated with the clinical phenotype of patients. As a noninvasive technology, NGS is capable of high-throughput sequencing with high sensitivity at relatively low cost, and it can effectively discover many new genes and pathways related to disease (Levy and Boone, 2019). In this study, we sought to analyze 15 cases of FUO utilizing WES to determine whether WES could be effectively utilized to identify the genetic cause of FUO.

## Materials and Methods

### Study Design and Participants

Fifteen patients hospitalized with FUO were selected in the First Affiliated Hospital of Zhejiang University between September 1, 2019 and September 1, 2021. All patients had fever lasting more than 3 weeks with a temperature over 38.3°C on several occasions, and the diagnosis was unclear after one week of inpatient investigation. Blood samples from these patients were collected for WES, and clinical, radiological, and pathogenic findings, as well as treatment data were extracted from the hospital information system. Ethics approval was obtained from the Institutional Review Board of the First Affiliated Hospital, Zhejiang University School of Medicine. Patients or their guardians signed a written informed consent.

### Sequencing

Genomic DNA was extracted from whole blood obtained from the patients and any participating family members. DNA samples were randomly disrupted by the ultrasonic high-performance sample processing system (Covaris). Segments were selected using the Agencourt AMPure XP-Medium kit, and library preparation was performed. Then the DNA of the target gene exon and adjacent splice regions was captured and enriched using a BGI V4 chip. Sequencing was performed on the Mgiseq-2000 sequencing platform. The average effective sequencing depth of the target region was ≥100X, and >95% of target bases, and had coverage >20X. The sequences were compared with the UCSC hg19 human reference genome by BWA. SNP detection and InDel analysis were performed, and then the mutation was selected by filtering through database annotation. The classification of variant pathogenicity is based on the American College of Medical Genetics (ACMG) guidelines.

## Results

These patients were between 15-63 years old, and they came to our hospital with different symptoms related to the respiratory system, cardiovascular system, immune system, central nervous system and skin. Seven patients were diagnosed with infectious diseases, including bronchiectasis with infection, Mycobacterium arhus infection, idiopathic pericarditis, Mycobacterium intracellular infection, Scedosporium boydii infection and Mycobacterium avium infection. Three patients were undiagnosed, and other patients were diagnosed as follows: pyoderma gangrenosum, hemophagocytic syndrome, systemic lupus erythematosus (SLE) and hemophagocytic syndrome, periodic fever syndrome, and pulmonary embolism. Patient characteristics are given in **Table 1**.

**Table 1:** Clinical characteristics of patients.

Sample	Age*/sex (M/F)	Diagnosis	Symptoms other than fever	Treatment	Outcome
S-1	63/F	Bronchiectasis with infection	cough and sputum	Anti-infection	Controlled without recurrence
S-2	26/M	Mycobacterium Aarhus infection	cough	Anti-mycobacterial therapy	Improved
S-3	26/M	Undiagnosed	/	Methylprednisolone	Controlled without recurrence
S-4	38/M	Pyoderma gangrenosum	damaged skin	Methylprednisolone	Improved
S-5	19/F	Undiagnosed	cough	NSAIDs; cough medicine	Controlled without recurrence
S-6	50/F	Idiopathic pericarditis	chest distress	Colchicines, Methylprednisolone	Improved
S-7	30/M	Mycobacterium intracellular infection	abdominal pain	Anti-mycobacterial therapy	Cured
S-8	23/F	Hemophagocytic syndrome	rash, joint pain	Methylprednisolone; Ciclosporin	Under treatment
S-9	54/M	Scedosporium boydii infection	headache	Voriconazole	Improved
S-10	21/F	Systemic Lupus Erythematosus; Hemophagocytic syndrome	cough	Etoposide; Ciclosporin	Under treatment
S-11	38/F	Periodic Fever Syndrome	joint pain	NSAIDs	Controlled without recurrence
S-12	47/M	Pulmonary embolism	cough, chest distress	Low molecular heparin, anti-infection	Cured
S-13	48/F	Mycobacterium intracellular infection	lymphadenectasis	Anti-mycobacterial therapy	Cured
S-14	26/F	Undiagnosed	headache	Symptomatic treatment	Under treatment
S-15	15/F	MonoMAC syndrome; Mycobacterium avium infection	cough and sputum	Anti-mycobacterial therapy	Under treatment

M, male; F, female; NSAIDs, nonsteroidal anti-inflammatory drugs. The symbol "*" means age of participant at initial referral.

We analyzed 15 cases of FUO using WES, with 14 submitted as probands only and 1 with two additional family members. The results were divided into three categories: definitive result refers to the pathogenic or likely pathogenic variant in a disease gene associated with the clinical phenotype, possible/probable diagnosis refers to variants in a disease gene possibly related to the clinical phenotype, negative result means that no variants in genes related to the reported phenotype have been found (Retterer et al., 2016). Overall, across the 15 cases, a definitive result was given in 2 cases, a possible/probable result was given in 5 (**Table 2**) and a negative result was given in 8 cases. The heterozygous missense mutation (c.2042A>T p.G lu681Val) in the *CFTR* gene in S-1 and the mutation (c.194_195insT p.Ala66Argfs*119) in the *GATA2* gene in S-15 were classified as likely pathogenic through ACMG guidelines for pathogenicity. The heterozygous missense mutation (c.1049-18A>G) in the *IRF2BP2* gene in S-2, the mutation (c.10601C>T p. Ser3534Leu) in the *ADGRV 1* gene in S-5, the compound heterozygous mutations (c.3083A>G p.Asn1028Ser, c.2590C>T p.Arg864Cys) in the *TYK2* gene in S-7, and the mutation (c.472T>G p.Cys158Gly) in the *THBD* gene in S-12 were all considered to be possibly associated with the reported phenotype. The pathogenicity of these mutations has not been reported and they were classified as a VoUS (variant of unknown significance) according to the ACMG guidelines. The mutation (c.220C>T p.Gln74*) in the *CD209* gene in S-2 and the heterozygous missense mutation (c.442G>C p.Glu148Gln) in the *MEFV* gene in S-11 have been reported previously. They were possibly associated with the reported phenotype and were also classified as a VoUS. WES data also identified two cases with CNVs greater than 1 Mb. A duplicate copy of chromosome 8 was detected in S-4, and approximately 60.8 Mb of suspected chimeric repeat was detected in the 1q21.1-q32.1 segment in S-6. This finding was not previously detected in the ISCA, Decipher and ClinVar databases and requires further study.

**Table 2:** The positive results of whole exon sequencing.

Sample	The primary results	Chromosomal location	Transcript ID: Mutation (amino acid variation)	ACMG	Relationship between mutations and phenotypes	Literature support
S-1	*CFTR*	chr7:117232263	NM_000492.3:c.2042A>T(p.G lu681Val)	Likely pathogenic	Partially related	Yes
S-2	*CD209*	chr19:7810932	NM_021155.3: c.220C>T(p.Gln74*)	VoUS	Partially related	Yes
	*IRF2BP2*	chr1:234743616	NM_182972.2: c.1049-18A>G		
S-5	*ADGRV 1*	chr5:90040914	NM_032119.3: c.10601C>T(p. Ser3534Leu)	VoUS	Partially related	Yes
S-7	*TYK2*	chr19:10463719	NM_003331.4: c.3083A>G(p.Asn1028Ser)	VoUS	Partially related	Yes
		chr19:10467271	NM_003331.4: c.2590C>T(p.Arg864Cys)		
S-11	*MEFV*	chr16:330425-3304 626	NM_000243.2: c.442G>C(p.Glu148Gln)	VoUS	Partially related	Yes
S-12	*THBD*	chr20:23029670	NM_000361.2: c.472T>G(p.Cys158Gly)	VoUS	Partially related	Yes
S-15	*GATA2*	chr3:128205680-128205681	NM_032638.4: c.194_195insT(p.Ala66Argfs*119)	Likely pathogenic	Partially related	Yes

ACMG, American College of Medical Genetics; VoUS, variant of unknown significance. The symbol "*" means nonsense mutations in amino acids.

## Discussion

We have performed clinical and molecular analyses in 15 patients with FUO to investigate the potential use of WES to identify genetic causes of FUO, which has rarely been studied before. The mutational analysis identified that 7 patients (46.7%) had positive results. The mutation detected in S-1 has previously been identified to play a role in bronchiectasis. *CFTR* can regulate airway epithelial surface mucus. Genetic defects of *CFTR* leads to poor mucus cilia clearance, excessive mucus secretion and recurrent infections with virulent pathogens including Pseudomonas aeruginosa, Staphylococcus aureus, and nontuberculous mycobacteria (Perrem and Ratjen, 2019). Studies have shown that heterozygosity for *CFTR* has pathogenic consequences and contributes to the development of bronchiectasis (Tzetis et al., 2001; Casals et al., 2004). This patient had been hospitalized several times with fever, cough and sputum, and had a history of intracellular mycobacterium infection. Therefore, the host factors that contributed to the disease were of great interest to us, and we look forward to finding better treatments. *CFTR* modulators are a new class of drugs that directly solve functional protein defects, and can enhance *CFTR* gene or protein expression levels at the cell surface (Patel et al., 2020). Significant progress has been made in the development, which is expected to improve the prognosis of patients.

S-2 carried two heterozygous missense mutations. The first was in the *CD209* gene (c.220C>T p.Gln74*) and the second was in the *IRF2BP2* gene (c.1049-18A>G). The *CD209* gene encodes the dendritic cell-specific intercellular adhesion molecule-3 binding nonintegrin factor (DC-SIGN) molecule, which is located on the surface of dendritic cells and alveolar macrophages. It is a natural immune recognition receptor associated with tuberculosis. Mycobacterium tuberculosis can bind to DC-SIGN through its cell wall component lipid arabinomannan (mannose-cappedlipoarabinomannan, ManLAM), leading to the infection of dendritic cells and alveolar macrophages (Barreiro et al., 2006). Studies have shown that *CD209* gene variants may affect the protection against and susceptibility to Mycobacterium tuberculosis infection (da Silva et al., 2014). Keller et al. showed that *IRF2BP2* variant led to failure of B cell differentiation, impaired immunoglobulin secretion and a familial form of common variable immunodeficiency disorder (CVID14) (Keller et al., 2016). The patient was young and had no structural bronchial abnormalities or other risk factors. Therefore, we highly suspect that these mutations compromised immune function and susceptibility to Mycobacterium tuberculosis in this patient.

A novel heterozygous mutation (c.10601C>T p. Ser3534Leu) in *ADGRV 1* was identified in S-5. *ADGRV1* mutation has been seen previously associated with familial febrile seizure 4 (Han et al., 2020), which is a febrile disease. Since the patient in our study also presented with recurrent fever without evidence of infection or common autoimmune disease, we propose that the new mutation in *ADGRV1* may have an impact on the occurrence of febrile disease in this patient. Further research is needed to prove that *ADGRV1* mutation is a significant susceptible gene for febrile disease.

**Figure 1:**
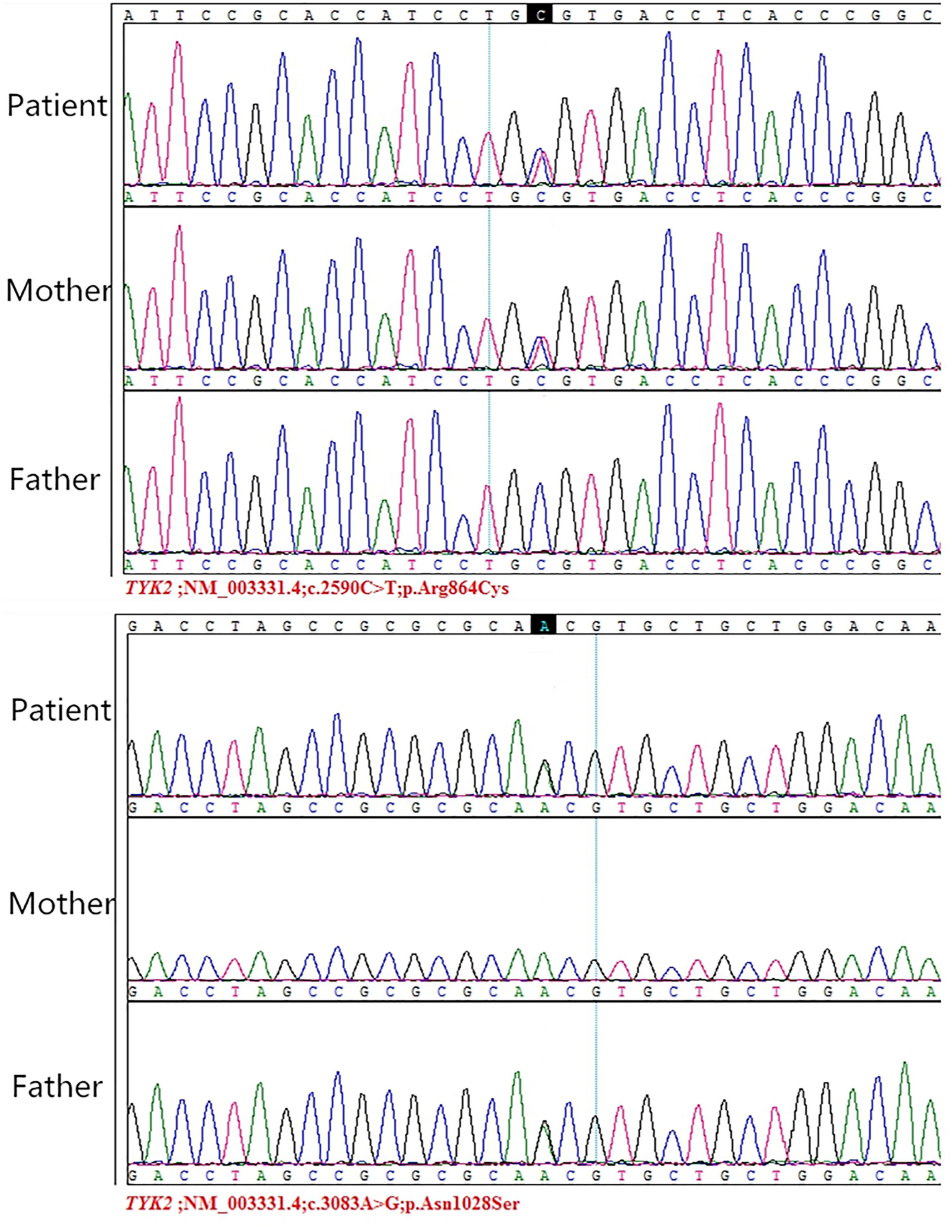
Sanger sequencing of TYK2 mutations in the patient and his parents. Reprinted with permission from Guo, W., Feng, X., Yang, M., Shangguan, Y., Shi, P., Wang, S., et al. (2020). Mycobacterium Intracellulare Infection Associated with TYK2 Deficiency: A Case Report and Review of the Literature. Infect. Drug. Resist. 13, 4347-4353. Creative Commons Attribution - Non Commercial (unported, v3.0) License (https://creativecommons.org/licenses/by-nc/3.0/).

WES revealed compound heterozygous mutations (c.3083A>G p.Asn1028Ser, c.2590C>T p.Arg864Cys) in the *TYK2* gene in S-7. Sanger sequencing showed that both parents were heterozygous for mutations in *TYK2*: c.2590C>T in the mother and c.3083A>G in the father (**Figure 1**). The patient had recurrent fever with nontuberculous mycobacteria invading the bowel, lymph nodes and bronchus. Previous studies have shown that *TYK2* mutations are associated with susceptibility to mycobacterial disease (Kreins et al., 2015; Rosain et al., 2019), which is consistent with this patient. In addition to conventional anti-tuberculosis treatment, injection of recombinant IFN-γ and transduction of the TYK2 gene are new treatment options (Minegishi et al., 2006; Bustamante et al., 2014).

The heterozygous missense (c.442G>C p.Glu148Gln) in the *MEFV* gene in S-11 has been reported to cause disease. Homozygous or compound heterozygous mutations can manifest as normal phenotypes and atypical partial clinical phenotypes of familial Mediterranean fever (FMF), while patients with typical FMF phenotypes are extremely rare (Cekin et al., 2017). These mutations may be conditional disease-causing polymorphic sites. Carrying multiple mutation sites may cause disease, but the condition may be atypical. S-11 presented with recurrent fever with joint pain, and periodic fever syndrome was considered, which is related to the *MEFV* mutation. Colchicine can effectively control FMF, and thalidomide, sulfasalazine, nonsteroidal anti-inflammatory drugs, and biological agents such as IL-1 antagonists and TNF-α inhibitors can also be used (Petrushkin et al., 2016).

S-12 carried a novel mutation (c.472T>G p.Cys158Gly) in the *THBD* gene. Thrombomodulin (THBD) is encoded by the *THBD* gene and expressed in vascular endothelial cells. It is a cofactor for thrombin-mediated activation of the anticoagulant protein C pathway and plays a key role in maintaining the balance of coagulation and anticoagulation (Esmon and Owen, 1981). Studies show that *THBD* mutations can lead to thrombomodulin protein defects, a tendency toward thrombosis, and an increased risk of venous thrombosis (Key and Reiner, 2016; Zhang et al., 2021), which supports the diagnosis of pulmonary embolism in this patient. The patient was admitted to the hospital due to fever, had a history of pulmonary embolism, and without risk factors such as trauma, tumor, long-term bed rest, and varicose veins of the lower extremities. Repeated attacks of pulmonary embolism were harmful to the patient, and the results of WES provide a basis for subsequent prevention. We think that individuals with *THBD* gene defects need to be more vigilant about the risk of thrombosis and take more active preventive and treatment measures.

S-15 carried a novel mutation (c.194_195insT p.Ala66Argfs*119) in the *GATA2* gene. The patient presented with fever of unknown origin, accompanied by cough and sputum. Blood cell count revealed monocytopenia (monocyte count: 10 cells/mm^3^). A transbronchial biopsy of the lung showed Mycobacterium avium infection. Pulmonary alveolar proteinosis was also seen in this patient. The combination of these phenomena supported the diagnosis of monocytopenia and mycobacterial infection (MonoMAC) syndrome caused by *GATA2* gene mutations, with nontuberculous mycobacterial infection as the main clinical feature. *GATA2* encodes a hematopoietic transcription factor that controls myeloid differentiation and plays an important role in the proliferation and differentiation of hematopoietic stem cells and multipotential progenitor cells (Ding et al., 2017). *GATA2* mutations not only lead to the reduction of monocytes, NK, B and dendritic cells, but also cause patients to be susceptible to MDS and AML (Camargo et al., 2013). Currently, symptomatic treatment is generally adopted according to the clinical manifestations of patients. Allogeneic hematopoietic stem cell transplantation has been proven to be an effective strategy to restore the patient’s hematopoietic and immune functions (Moraes-Fontes et al., 2019).

According to the current literature and related studies, no proven phenotype-related mutations were detected in the remaining eight samples, which may be caused by other mutation sites. As our data accumulate, further investigation of FUO patients in a larger patient cohort is likely to identify genetic mutations not included in the current study. Two of the eight patients were diagnosed as Scedosporium boydii infection and mycobacterium intracellular infection respectively, whose FUO phenotypes was thought to be related to pathogens/pathogen-derived factors.

The diagnosis of FUO remains challenging. Many FUOs caused by inflammatory diseases and rare diseases are particularly difficult to diagnose and require a high degree of clinical suspicion as well as reliable molecular evidence to confirm. Advances in molecular genetic technology have increased the importance of these tests in the diagnosis of FUO. Based on our experience, we recommend WES as an auxiliary test for many indications, including no immunodeficiency found in routine examination, such as HIV, complement, antibodies, and cytokines, but clinically highly suspected immune abnormalities and, suspicion of self-inflammatory disease and deficiencies in inflammation regulation. Importantly, some diagnostic results can affect the treatment options and improve the prognosis. New treatments that target genetic mutations may be more effective than traditional treatments. We believe that WES will play an increasingly important role in the diagnosis and treatment of FUO-related infectious and noninfectious diseases. Despite the rapid development of WES technology, it also has some limitations. First, structural mutations, large fragment insertion mutations, dynamic mutations and complex recombination mutations cannot be detected. Second, the database it uses is not perfect, so the analysis results may not be comprehensive. Additionally, the report takes approximately four weeks, resulting in the lack of timely guidance for disease diagnosis and treatment. Thus, clinical data needs to be further accumulated in future studies. We hope that more global multicenter clinical data and WES results can be uploaded simultaneously, so that more genetic-related diseases can be recognized.

Our research has some limitations. The sample size in our study was modest; thus, larger studies are needed to analyze the role of WES in patients with FUO in the future. We also have not further confirmed the pathogenicity of these mutations discovered by WES and cannot determine whether these mutations are etiologies of these patients. Subsequent separation and functional studies at the PBMC level other than the whole blood level are needed to support our findings.

## Conclusion

Here, we demonstrate the application of WES in patients with FUO to identify potential disease-causing genetic mutations. Through this analysis, we found positive results in 7 patients, including 8 gene variants, representing a positive rate of 46.7% in our population (n=7/15). In conclusion, WES successfully detected mutant genes and identified some potential diseases causing fever, which has a complementary role in the diagnosis and treatment of patients with FUO.

## Data Availability Statement

The original contributions presented in the study are included in the article/supplementary material. Further inquiries can be directed to the corresponding author.

## Ethics Statement

The studies involving human participants were reviewed and approved by the Ethics Committee of the First Affiliated Hospital, Zhejiang University School of Medicine. Written informed consent to participate in this study was provided by the participants’ legal guardian/next of kin. Written informed consent was obtained from the individual(s), and minor(s)’ legal guardian/next of kin, for the publication of any potentially identifiable images or data included in this article.

## Author Contributions

WG, XF, MH, YaS, and KX was involved in the conception and design of the study. WG, MH, YaS, JX, WH, XL, ZZ, and YuS were involved in the collection and assembly of data. WG, XF, and MH were involved in interpreting the data. WG and XF wrote the manuscript. All authors contributed to the article and approved the submitted version.

## Funding

This work was supported by grants from the Regional Characteristics Of Nontuberculous Mycobacteria In Lishui (grant number 2018zdhz10).

## Conflict of Interest

The authors declare that the research was conducted in the absence of any commercial or financial relationships that could be construed as a potential conflict of interest.

## Publisher’s Note

All claims expressed in this article are solely those of the authors and do not necessarily represent those of their affiliated organizations, or those of the publisher, the editors and the reviewers. Any product that may be evaluated in this article, or claim that may be made by its manufacturer, is not guaranteed or endorsed by the publisher.
